# Klebsiella pneumoniae Infection as a Mimicker of Multiple Metastatic Lesions

**DOI:** 10.7759/cureus.32669

**Published:** 2022-12-18

**Authors:** Ayushi Shah, Akshay Shetty, David Victor, Sudha Kodali

**Affiliations:** 1 Internal Medicine, Houston Methodist Hospital, Houston, USA; 2 Hepatology and Transplant Medicine, Houston Methodist Hospital, Houston, USA

**Keywords:** septic pulmonary emboli, bacterial pyomyositis, sepsis in liver cirrhosis, metastatic infection, klebsiella pneumoniae (kp)

## Abstract

We describe the case of a 42-year-old man with cirrhosis who presented with fever and imaging concerning for metastatic disease from suspected renal cell carcinoma. He had a right renal mass with multiple pulmonary masses and underwent a lung biopsy and oncology consultation. Blood cultures revealed *Klebsiella pneumoniae*, and all the lesions disappeared after intravenous (IV) antibiotics. Our case attempts to increase awareness of this unique presentation of invasive *Klebsiella *infections and discusses host factors that can predispose to this condition.

## Introduction

Infection and ascites are one of the most common causes of hospitalization in cirrhotic patients. Immune dysfunction, gut dysbiosis, and increased bacterial translocation that occurs in cirrhosis can predispose these patients to invasive infections with increased mortality [1]. Gram-negative Enterobacteriaceae from the intestinal origin are frequently isolated as a source of infection in cirrhotic patients, while gram-positive bacteria are a frequent cause of infection in hospitalized patients [1]. We describe a rare case of *Klebsiella pneumoniae* infection in a patient with cirrhosis that at the time of presentation was concerning for diffusely metastatic disease with suspected primary renal cell carcinoma. 

## Case presentation

A 42-year-old male with a history of decompensated alcohol-related cirrhosis was admitted to the hospital with acute onset of fever, jaundice, and poor oral intake for two weeks. He also reported diarrhea for five days. His past medical history included hypertension and anxiety. He reported drinking two bottles of wine daily. Physical examination showed that he was alert and oriented, with a temperature of 99.1 °F, heart rate of 100 beats per minute, and blood pressure of 152/90 mmHg. He had tenderness over the right costovertebral angle, with a soft, nontender abdomen. There was no flank dullness to suggest ascites. Laboratory results are summarized in Table 1.

**Table 1:** Initial laboratory results obtained on admission. mEq/L, milliequivalents per liter; mg/dL, milligrams per deciliter; U/L, units per liter; mmol/L, millimoles per liter; μL, microliter; fL, femtoliter; GFR, glomerular filtration rate; ALT, alanine transaminase; AST, aspartate aminotransferase; INR, international normalized ratio; WBC, white blood cell

Laboratory test	Result	Reference ranges
Sodium (mEq/L)	110	135-148
Potassium (mEq/L)	4.5	3.5-5
Chloride (mEq/L)	88	98-112
CO_2_ (mEq/L)	22	24-31
Anion gap (mEq/L)	9	7-15
Blood urea nitrogen (mg/dL)	19	6-20
Creatinine (mg/dL)	0.91	0.7-1.2
Estimated GFR (mL/min/1.73 m^2^)	>=90	>=90
Glucose (mg/dL)	134	65-99
Protein (g/dL)	6.1	6.3-8.3
Albumin (g/dL)	1.8	3.5-5
Alkaline phosphatase (U/L)	148	40-129
ALT (U/L)	46	5-50
AST (U/L)	76	10-50
Total bilirubin (mg/dL)	8	0.0-1.2
Lactic acid (mmol/L)	2.9	0.5-2.2
WBC count (μL^-1^)	26,190	4,500-11,000
Hemoglobin (g/dL)	10.3	14-18
Hematocrit (%)	28.60	41-51
Mean corpuscular volume (fL)	93.2	82-100
Platelet count (μL^-1^)	63,000	150,000-400,000
Prothrombin time (seconds)	28.8	11.5-14.5
INR	2.7	

Urinalysis showed pyuria with nitrites and hematuria as summarized in Table 2. 

**Table 2:** Urinalysis. RBC, red blood cell; WBC, white blood cell; HPF, high-power field

Urinalysis	Result	Reference range
Color	Amber	-
Appearance	Cloudy	-
Glucose (urinalysis)	Negative	Negative
Bilirubin (urinalysis)	Negative	Negative
Ketones (urinalysis)	Trace	Negative
Specific gravity	1.013	1.001-1.035
Blood (urinalysis)	Moderate	Negative
pH	5	5-8.5
Protein	Negative	Negative
Urobilinogen	4	<2
Nitrite	Positive	Negative
Leukocyte esterase	Moderate	Negative
RBC (urinalysis)	8/HPF	0-1/HPF
WBC	71/HPF	0-5/HPF
Bacteria	Many	Few
Yeast	None seen	
Yeast with pseudohyphae	None seen	

Viral hepatitis serologies were negative. The Model for End-Stage Liver Disease-Na (MELD-Na) score was 31. Two sets of blood cultures grew *K. pneumoniae* in both aerobic and anaerobic bottles. Urine culture grew more than 100,000 colony-forming units of *K. pneumoniae*. The susceptibility of the organism isolated from blood and urine cultures is described in Table 3.

**Table 3:** Antibiotic susceptibility of Klebsiella pneumoniae. MIC, minimum inhibitory concentration

Antibiotic name	*Klebsiella pneumoniae* MIC	Susceptibility
Amikacin	<=8 mcg/mL	Susceptible
Amoxicillin/Clavulanate	<=4/2 mcg/mL	Susceptible
Ampicillin	>16 mcg/mL	Resistant
Ampicillin/Sulbactam	8/4 mcg/mL	Susceptible
Aztreonam	<=2 mcg/mL	Susceptible
Cefazolin	<=1 mcg/mL	Susceptible
Cefepime	<=1 mcg/mL	Susceptible
Cefoxitin	<=4 mcg/mL	Susceptible
Ceftaroline	<=0.25 mcg/mL	Susceptible
Ceftazidime	<=2 mcg/mL	Susceptible
Ceftriaxone	<=1 mcg/mL	Susceptible
Cefuroxime sodium	<=4 mcg/mL	Susceptible
Ciprofloxacin	<=0.25 mcg/mL	Susceptible
Ertapenem	<=0.25 mcg/mL	Susceptible
Gentamicin	<=2 mcg/mL	Susceptible
Levofloxacin	<=0.5 mcg/mL	Susceptible
Meropenem	<=0.5 mcg/mL	Susceptible
Piperacillin/Tazobactam	4/4 mcg/mL	Susceptible
Tetracycline	<=2 mcg/mL	Susceptible
Tigecycline	<=1 mcg/mL	Susceptible
Tobramycin	<=2 mcg/mL	Susceptible
Trimethoprim/Sulfamethoxazole	<=0.5/9.5 mcg/mL	Susceptible

**Figure 1:**
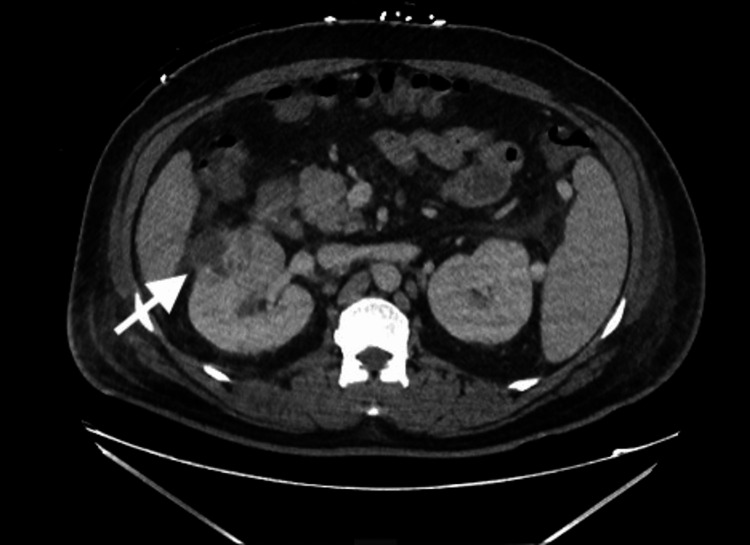
CT abdomen and pelvis showing a 4 cm heterogeneously enhancing right renal mass suspicious for renal cell carcinoma.

CT of the abdomen and pelvis was interpreted by radiology as a 4 cm heterogeneous enhancing right renal mass, suspicious for renal cell carcinoma, as shown in Figure 1. 

**Figure 2:**
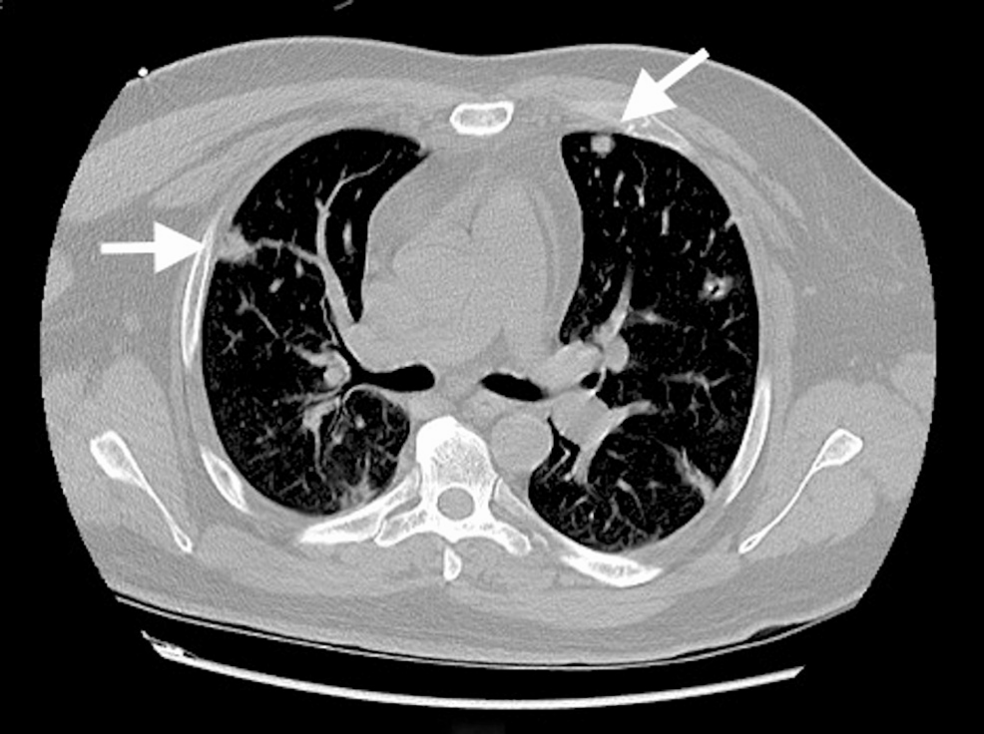
CT chest showing multiple bilateral pulmonary nodules highly suspicious for metastatic disease, measuring up to 1.5 cm in the right upper lobe.

Consequently, a CT chest was obtained, which demonstrated multiple pulmonary nodules bilaterally up to 1.5 cm and mediastinal lymphadenopathy, as shown in Figure 2.

**Figure 3:**
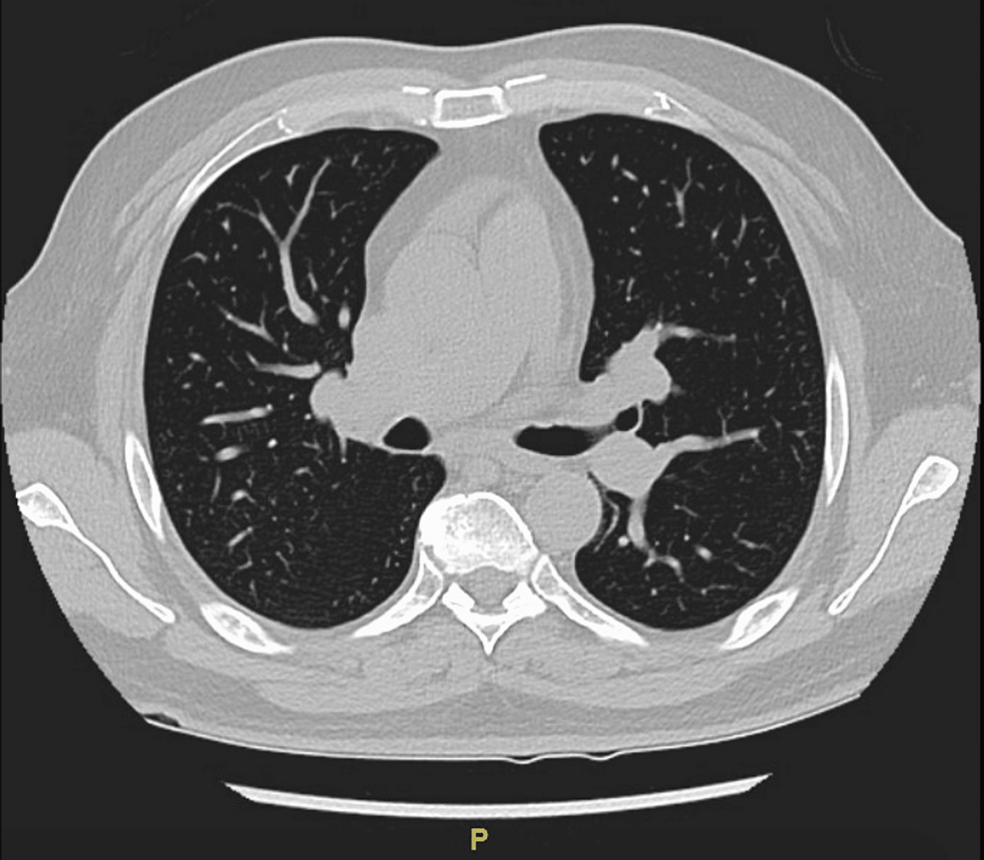
Resolution of the pulmonary ground glass opacities after IV antibiotics. IV, intravenous

**Figure 4:**
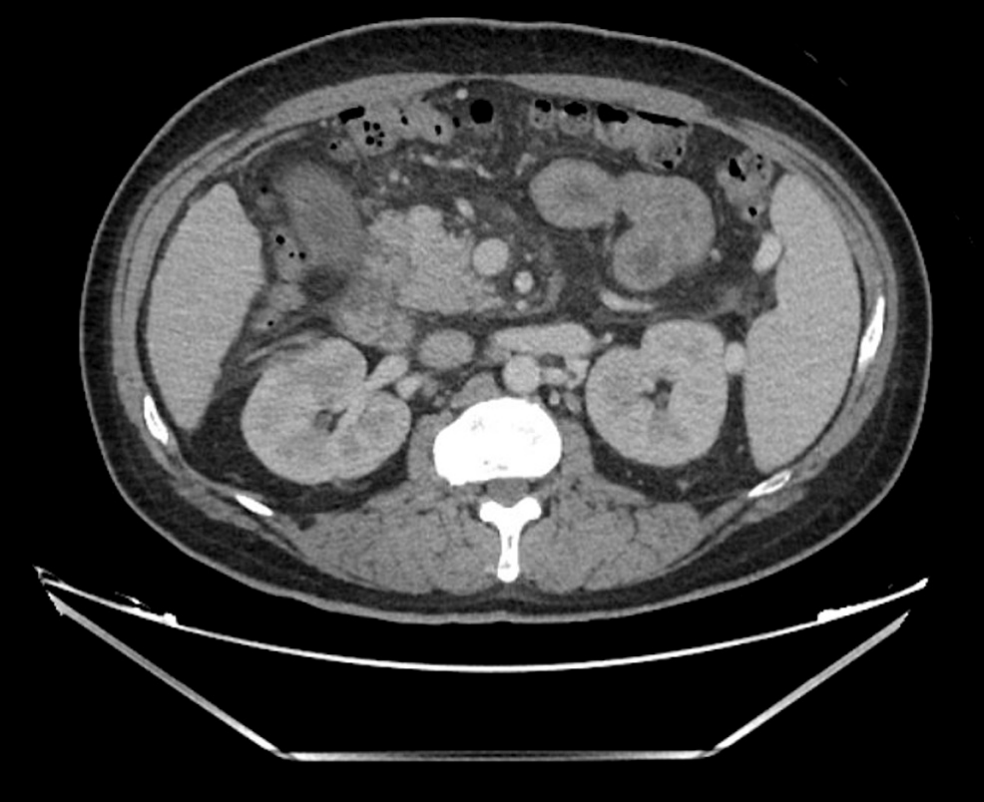
Resolution of renal abscess after IV antibiotics. IV, intravenous

Given the radiological appearance of these masses, there was a strong suspicion that these were metastatic masses from a primary renal malignancy. Tumor markers were ordered, and oncologists were consulted. A renal biopsy was planned for histopathological diagnosis; however, after weighing the risks of post-procedural bleeding between a renal and lung biopsy, a CT-guided percutaneous needle biopsy of a right upper lobe pulmonary nodule was pursued and showed necroinflammation suggestive of an abscess. Before the biopsy, the coagulopathy was addressed with a transfusion of fresh frozen plasma and intravenous vitamin K after which the international normalized ratio (INR) was 1.6 on the day the biopsy was conducted. Cytology was negative for malignancy. The patient received two weeks of IV ceftriaxone 2 g every 24 hours, and repeat imaging of the abdomen showed a reduction in the size of the renal mass to 2.5 cm with features now consistent with perinephric abscess. The radiologic features seen on CT this time included an ill-defined parenchymal abnormality in the anterior right kidney, which was overall improving and consistent with evolving pyelonephritis with a stable 2.5 cm overlying perinephric abscess. Drainage was not pursued at this time as its size was less than 3 cm, and it was responding to antibiotics. Similarly, the pulmonary nodules had reduced in size and number in response to the antibiotics, thus revealing their infectious nature. While on IV ceftriaxone for two weeks, the patient then developed septic arthritis of the right sternoclavicular joint with pyomyositis of the right pectoralis muscles. Drainage of the muscle abscess also grew the same isolate of *Klebsiella *as identified in the blood and urine cultures. An echocardiogram to evaluate for infective endocarditis was negative. Repeat blood cultures done 3 and 14 days after the initial blood culture were negative. He received IV ceftriaxone for eight weeks. A follow-up CT scan of the chest and abdomen three months after discharge (Figures 3, 4) showed complete resolution of the renal abscess, myositis, and pulmonary nodules with residual scarring in the lungs. 

## Discussion

This case study attempts to increase awareness of an invasive *Klebsiella* infection that can lead to multiple septic metastases, which can be mistaken for malignancy before invasive testing, as mentioned above. Most community-acquired *Klebsiella *infections are associated with pneumonia or urinary tract infections. In the past few decades, an invasive syndrome called *K. pneumoniae* invasive syndrome, which causes primary liver abscesses followed by metastatic abscesses, has been reported. Our case is a rare presentation, where our patient had widespread dissemination without the presence of any hepatic abscesses [2]. This is a well-known entity in South-East Asia, but more recently, it has also been reported in other continents [3]. Specific hypervirulent capsular strains (K1 and K2) and diabetes mellitus predispose the host to an invasive infection [4-6]. We do not know the capsular serotype of this infection in our patient as typing was not available at our institution. This patient’s risk factors for invasive infection included heavy alcohol use, protein-calorie malnutrition, and cirrhosis-associated immune dysfunction [7]. Cirrhosis of the liver is associated with immune dysfunction due to damage to the reticuloendothelial system, deficient bactericidal and opsonization capabilities related to low levels of C3 complement protein, and impairment in chemotaxis mechanisms [7,8].

In patients with cirrhosis, these infections are associated with spontaneous bacterial peritonitis, urinary tract infections, and community-acquired pneumonia [6]. The incidence of *Klebsiella* infections in patients with cirrhosis is variable. In a prospective study of 159 cirrhotic patients in India, 23% of infections were secondary to *K. pneumoniae* [9]. Another study in Spain reported an incidence of *K. pneumoniae* infections in patients with cirrhosis as 1.9% [10]. This patient developed septic arthritis and pyomyositis despite two weeks of antibiotics, which further underscores the increased risk of invasive infections in cirrhotic patients. Hypoproteinemia, ascites, third space expansion, and impaired renal function have been postulated to cause unpredictable drug exposure, response, and altered pharmacokinetics of antimicrobials [11].

In our patient, the imaging-based presumption of neoplasia could have led to incorrect and unnecessary investigations, thus highlighting the importance of increasing awareness of septic metaplasia secondary to certain *Klebsiella* infections. In a systematic review of 168 patients with septic pulmonary emboli, 8% of these cases were secondary to *Klebsiella* [12]. Septic pulmonary emboli with metastatic infection to other vital organs pose a poor prognosis [2]. Joint involvement is quite rare as evidenced by the few cases reported in the literature [13]. Of these cases, diabetes, heavy alcohol use, and cirrhosis or IV drug use have been the major risk factors for hematogenous spread to bone or joint, as seen in this patient [13].

We weighed the risks versus benefits of doing a lung biopsy compared to a renal biopsy and proceeded with a lung biopsy after correcting his coagulopathy due to the lower risk of post-procedural bleeding [14,15].

Community-acquired *Klebsiella* isolates remain susceptible to cephalosporins. The treatment regimen involves third- or fourth-generation cephalosporins and varies from two to four weeks for a solitary abscess and at least six weeks for multiple abscesses [16].

## Conclusions

Our case study demonstrates metastatic *Klebsiella* infection with multiple pulmonary septic emboli, a pyogenic renal abscess, and muscle involvement that was initially suspicious for a metastatic malignancy due to the radiographic appearance of the right renal mass and the presence of multiple lung lesions. Widespread dissemination of *Klebsiella* with abscesses is possible while sparing the liver. Early recognition and prompt treatment are important as this infection is associated with a high rate of morbidity and mortality.
